# Regulated intramembrane proteolysis of the AXL receptor kinase generates an intracellular domain that localizes in the nucleus of cancer cells

**DOI:** 10.1096/fj.201600702R

**Published:** 2016-12-29

**Authors:** Yinzhong Lu, Jun Wan, Zhifeng Yang, Xiling Lei, Qi Niu, Lanxin Jiang, Willemijn M. Passtoors, Aiping Zang, Patrick C. Fraering, Fang Wu

**Affiliations:** * Key Laboratory of Systems Biomedicine, Ministry of Education, Shanghai Center for Systems Biomedicine, Shanghai Jiao Tong University, Shanghai, China;; † School of Life Science and Biotechnology, Shanghai Jiao Tong University, Shanghai, China;; ‡ Brain Mind Institute–School of Life Sciences, Ecole Polytechnique Fédérale de Lausanne, Lausanne, Switzerland;; § Foundation Eclosion, Plan-Les-Ouates, Switzerland;; ¶ Campus Biotech Innovation Park, Geneva, Switzerland

**Keywords:** receptor tyrosine kinase, presenilin, γ-secretase, protein processing, nuclear translocation

## Abstract

Deregulation of the TAM (TYRO3, AXL, and MERTK) family of receptor tyrosine kinases (RTKs) has recently been demonstrated to predominately promote survival and chemoresistance of cancer cells. Intramembrane proteolysis mediated by presenilin/γ-secretase is known to regulate the homeostasis of some RTKs. In the present study, we demonstrate that AXL, but not TYRO3 or MERTK, is efficiently and sequentially cleaved by α- and γ-secretases in various types of cancer cell lines. Proteolytic processing of AXL redirected signaling toward a secretase-mediated pathway, away from the classic, well-known, ligand-dependent canonical RTK signaling pathway. The AXL intracellular domain cleavage product, but not full-length AXL, was further shown to translocate into the nucleus *via* a nuclear localization sequence that harbored a basic HRRKK motif. Of interest, we found that the γ-secretase–uncleavable AXL mutant caused an elevated chemoresistance in non–small-cell lung cancer cells. Altogether, our findings suggest that AXL can undergo sequential processing mediated by various proteases kept in a homeostatic balance. This newly discovered post-translational processing of AXL may provide an explanation for the diverse functions of AXL, especially in the context of drug resistance in cancer cells.—Lu, Y., Wan, J., Yang, Z., Lei, X., Niu, Q., Jiang, L., Passtoors, W. M., Zang, A., Fraering, P. C., Wu, F. Regulated intramembrane proteolysis of the AXL receptor kinase generates an intracellular domain that localizes in the nucleus of cancer cells.

Receptor tyrosine kinases (RTKs) are known to localize to the plasma membrane and transmit signals *in situ* *via* intracellular signaling cascades (1); however, a few RTKs can be cleaved by γ-secretase, known as regulated intramembrane proteolysis (RIP) (1, 2). The resulting intracellular domain (ICD) is released from the membrane and translocates into the cytoplasm and/or nucleus (1). Although some ICDs from RTKs play important roles in physiological processes (3, 4), the molecular function of most ICDs remains unclear.

AXL is an RTK that composes the TAM (TYRO3, AXL, and MERTK) family, together with TYRO3 and MERTK. It is a single-pass, type I transmembrane protein that encodes 849 aa containing 2 Ig domains, 2 fibronectin III domains, and 1 cytosolic kinase domain. Gas6 is the only ligand that binds to AXL identified to date, with a higher affinity for AXL than for TYRO3 or MERTK (5). AXL is widely expressed in various tissues and has been shown to have diverse functions (for example, induction of drug resistance, clearance of apoptotic cells, or negative regulation of immune response) (6–8). The classic AXL signaling pathway is initiated with binding of Gas6 to AXL to induce the AXL receptor dimerization and ICD autophosphorylation. Then, it transduces extracellular signaling to the downstream effectors, including but not limited to PI3K/Akt, MAPK, or PKC (9), and ultimately exerts biological effects on cells or organisms (10).

AXL is overexpressed in various cancers, and its expression levels correlate with a low survival rate in such cancers as lung (11), pancreatic (12, 13), and breast cancer (14–16). Recently, overexpression of AXL has been demonstrated to cause drug resistance in various cancer cells (5, 6, 17). Pharmacological inhibition of the kinase activity of AXL has been demonstrated to counteract drug resistance in tyrosine kinase inhibitors (TKIs) *in vitro* and *in vivo*, which is an approach that is currently being tested in clinical trials for several types of cancers (9). Thus, AXL has emerged as a novel therapeutic target for cancer (18).

The protein level of full-length AXL (AXL-FL) is regulated by a disintegrin and metalloproteinase (ADAM) and ubiquitin ligase (19–22). Metalloproteases ADAM10 and tumor necrosis factor α converting enzyme (TACE) have been reported to cleave AXL and release ectodomain [that is, a soluble AXL (sAXL) of ∼80 kDa] (19, 22, 23). Recently, Casitas B-lineage lymphoma E3 ligase was also found to regulate the abundance of AXL in cells (21).

In this study, we hypothesized that TAM RTKs could be proteolytically cleaved by α- and γ-secretases in cells. We found that AXL-FL, but not MERTK or TYRO3, was very efficiently cleaved by α- and γ-secretases in various types of cancer cell lines. The cleavage product, AXL intracellular domain (AXL-ICD), which contained a nuclear localization sequence (NLS), was released from the plasma membrane and further translocated into both the cytoplasm and nucleus. Moreover, inhibition of proteasome activity could accelerate the newly discovered intramembrane proteolytic cleavage process of AXL, leading to reduced levels of AXL-FL in cells. Of importance, preventing cleavage of AXL seems to promote cell survival and resistance to erlotinib, a prescribed TKI drug of epidermal growth factor receptor, in non–small-cell lung cancer (NSCLC) cells.

## MATERIALS AND METHODS

### Reagents and Abs

TNF-α protease inhibitor 1 (TAPI-1) was purchased from Santa Cruz Biotechnology (Santa Cruz, CA, USA); C3 from Calbiochem (Billerica, MA, USA); DAPI and protein G-agarose beads from Roche (Basel, Switzerland); epoxomicin (Epo) from Cayman Chemical (Ann Arbor, MI, USA); and DMSO, phorbol 12-myristate 13-acetate (PMA), phosphatidylcholine (PC), and Chapso from Sigma-Aldrich (St. Louis, MO, USA). Anti-DYKDDDDK affinity gel was from Biotool (Shanghai, China); and *N*-[*N*-(3,5-difluorophenacetyl)-L-alanyl]-*S*-phenylglycine *t*-butyl ester (DAPT), RO4929097, and R428 (BGB324) from Selleck (Houston, TX, USA). Abs used in this study were as follows: C-terminal AXL polyclonal Ab [C-20; for AXL, AXL-CTF (AXL C-terminal fragment), or AXL-ICD, Western blot (WB): 1:200; sc-1096; Santa Cruz Biotechnology], AXL-CTF mAb (B-2; for AXL, AXL-CTF, or AXL-ICD, WB: 1:200; sc-166268; Santa Cruz Biotechnology), pan-pY (4G10; WB: 1:1000; Millipore, Billerica, MA, USA), ab76083 (mAb for PS1-CTF, WB: 1:1000; Abcam, Cambridge, MA, USA), anti-ADAM10 (WB:1:1000, ab1997; Abcam), LMNB1 (WB: 1:500, 12987-1-AP; Proteintech Group, Chicago, IL, USA), and TACE (WB:1:1000, 6978S; Cell Signaling Technologies, Beverly, MA, USA). Abs that targeted β-actin (WB: 1:2000, M20010), β-tubulin (WB: 1:2000, M30109), Flag-tag (WB: 1:2000, immunofluorescence (IF): 1:100, M20008), Myc-tag (WB: 1:2000, M20002), Histone H3.1 (WB: 1:5000, P30266), and glyceraldehyde 3-phosphate dehydrogenase (GAPDH); WB: 1:2000, M20006) were purchased from Abmart (Shanghai, China). Secondary Abs conjugated with Alexa Fluor 680 [that is, goat anti-rabbit IgG (WB: 1:2000, A-21109), goat anti-mouse (WB: 1:2000, A-21058), and donkey anti-goat IgG (WB: 1:2000, A-21084)] or Alexa Fluor 546 anti-mouse secondary Ab (IF: 1:2000, A-11030) were purchased from Thermo Fisher Scientific (Waltham, MA, USA).

### Plasmids

All plasmids and primers used in cloning experiments described below are summarized in **Table 1** and Supplemental Table 1, respectively. Human TAM cDNAs were purchased from Proteintech Group or Genechem (Shanghai, China). AXL-FL cDNA was amplified by PCR with primers 1 and 2 or with primers 3 and 4 and cloned with a C-terminal Flag-tag into plasmids pcDNA3 (Thermo Fisher Scientific) and pCDH-CMV (Systems Biosciences, Mountain View, CA, USA), respectively. MERTK and TYRO3 were cloned into pcDNA3 with primers 5 and 6 and 7 and 8, respectively, to generate C-terminal Flag-tagged proteins. AXL_(428-527)_ and AXL_(445-527)_ were subcloned by using the pcDNA3-AXL vector as a template into the pET21b vector to express recombinant proteins by using primers 9 and 11 and 10 and 11, respectively. pCDH-AXL-ICD-Flag_(473-894)_ was generated by using the pCDH-AXL-Flag as a template and primers 12 and 4.

**TABLE 1.** DNA templates and primers for cloning

Construct	DNA template	Primers	Vector
pcDNA3-AXL-Flag	AXL cDNA	1, 2	pcDNA3
pCDH-AXL-Flag	AXL cDNA	3, 4	pCDH-CMV
pcDNA3-MERTK-Flag	MERTK cDNA	5, 6	pcDNA3
pcDNA3-TYRO3-Flag	TYRO3 cDNA	7, 8	pcDNA3
pET21b-AXL_(428-527)_-Flag	AXL cDNA	9, 11	pET21b
pET21b-AXL_(445-527)_-Flag	AXL cDNA	10, 11	pET21b
pCDH-AXL_(473-894)_-Flag	AXL cDNA	12, 4	pCDH-CMV
Δ1 (AXL428-435)	pCDH-AXL-Flag	13, 14	pCDH-CMV
Δ2 (AXL436-443)	pCDH-AXL-Flag	15, 16	pCDH-CMV
Δ3 (AXL444-451)	pCDH-AXL-Flag	17, 18	pCDH-CMV
L437A	pcDNA3-AXL-Flag	19, 20	pcDNA3
V438A	pcDNA3-AXL-Flag	21, 22	pcDNA3
L437A/V438A	pcDNA3-AXL-Flag	23, 24	pcDNA3
mut1	pCDH-AXL-Flag	25, 26	pCDH-CMV
mut2	pCDH-AXL-Flag	27, 28	pCDH-CMV
mut3	pCDH-AXL-Flag	29, 30	pCDH-CMV
mut4	pCDH-AXL-Flag	31, 32	pCDH-CMV
mut1a	pCDH-AXL-Flag	33, 34	pCDH-CMV
mut1b	pCDH-AXL-Flag	35, 36	pCDH-CMV
mut1c	pCDH-AXL-Flag	37, 38	pCDH-CMV
V459A	pcDNA3-AXL-Flag	39, 40	pcDNA3
V472A	pcDNA3-AXL-Flag	41, 42	pcDNA3
V459Δ	pcDNA3-AXL-Flag	43, 44	pcDNA3
V472Δ	pcDNA3-AXL-Flag	45, 46	pcDNA3
V458L	pcDNA3-AXL-Flag	47, 48	pcDNA3
A468T	pcDNA3-AXL-Flag	49, 50	pcDNA3
V458L/A468T	V458L	49, 50	pcDNA3
K567R	pcDNA3-AXL-Flag	51, 52	pcDNA3
Flag-AXL	pCDH-AXL-Flag	53, 54	pCDH-CMV
AXL_(473-894)_-EGFP	AXL cDNA	55, 58	pEGFP-N2
AXL_(481-894)_-EGFP	AXL cDNA	56, 58	pEGFP-N2
AXL_(531-894)_-EGFP	AXL cDNA	57, 58	pEGFP-N2
AAAAA	AXL_(473-894)_-EGFP	59, 60	pEGFP-N2
AAAAK	AXL_(473-894)_-EGFP	61, 62	pEGFP-N2
HAAAA	AXL_(473-894)_-EGFP	63, 64	pEGFP-N2
AAKK	AXL_(473-894)_-EGFP	65, 66	pEGFP-N2
RAAK	AXL_(473-894)_-EGFP	67, 68	pEGFP-N2
RRAA	AXL_(473-894)_-EGFP	69, 70	pEGFP-N2
RRKA	AXL_(473-894)_-EGFP	71, 72	pEGFP-N2
RRAK	AXL_(473-894)_-EGFP	73, 74	pEGFP-N2
RAKK	AXL_(473-894)_-EGFP	75, 76	pEGFP-N2
ARKK	AXL_(473-894)_-EGFP	77, 78	pEGFP-N2
NF-κB-Luc	5× κB response element	79, 80	pGL3 Basic

Primer values correspond to no. in Supplemental Table 1.

For characterization of α-secretase cleavage sites in AXL, Δ1(^428^PGQAQPVH^435^-deletion), Δ2 (^436^QLVKEPST^443^-deletion), and Δ3 (^444^PAFSWPWW^451^-deletion), as well as single or double mutants L437A, V438A, and L437A/V438A, were generated from pcDNA3-AXL-Flag by PCR using primers 13–14, 15–16, 17–18, 19–20, 21–22, and 23–24, respectively. Similarly, mut1-4, mut1a, 1b, 1c, V459A, V472A, ΔV459, and ΔV472 were produced by using primers 25–26, 27–28, 29–30, 31–32, 33–34, 35–36, 37–38, 39–40, 41–42, 43–44, and 45–46, respectively, to investigate γ-secretase cleavage site in AXL. V458L, A468T, V458L/A468T, and K567R mutants were generated by using primers 47–48, 49–50, 47–50, and 51–52, respectively. To study the α-secretase cleavage site for AXL, we introduced an additional Flag-tag at the N terminus of AXL (Flag-AXL) with primers 53–54. For colocalization studies, truncated AXL_(473-894)_, AXL_(481-894)_, and AXL_(531-894)_ were amplified by using primers 55 and 58, 56 and 58, and 57 and 58, respectively, and subcloned into the plasmid carrying enhanced green fluorescent protein (pEGFP-N2; Clontech, Palo Alto, CA, USA) to generate an in-frame fusion with EGFP. AAAAA-, AAAAK-, HAAAA-, AAKK-, RAAK-, RRAA-, RRKA-, RRAK-, RAKK-, and ARKK-EGFP plasmids were constructed by using the AXL_(473-894)_-EGFP plasmid as a template and primers 59–60, 61–62, 63–64, 65–66, 67–68, 69–70, 71–72, 73–74, 75–76, and 77–78. All mutations were created with site-directed mutagenesis method according to Strategene’s instructions by using specific primers, KOD-plus-neo (Toyobo, Osaka, Japan) and Dpn I (New England BioLabs, Ipswich, MA, USA). All plasmids were confirmed by DNA sequencing.

Plasmid pRK5F-TACE (plasmid 31713) and pRK5M-ADAM10 (plasmid 31717) were obtained from Addgene (Cambridge, MA, USA) (24).

### Cell lines and cell culture

HCT116, HCC827, H1299, AGS, AsPC-1, Panc-1, and MDA-MB-231 cells were originally obtained from the Shanghai Cell Bank (Chinese Academy of Science, Shanghai, China). HEK293T, Hela, LN-18, and LN-229 cells were kind gifts from Dr. H. Gehring (University of Zurich, Zurich, Switzerland), Panc-28 cells were from Dr. X. Lin (Chinese Academy of Sciences, Qingdao, China), and SGC7901 and A549 cells were from Dr. Y. Zhang (Shanghai Jiao Tong University). WT, presenilin1-knockout (PS1-KO), PS2-KO, and PS1/2 double-KO, (PS-DKO) mouse embryonic fibroblasts (MEFs) were kindly provided by Dr. B. De Strooper (University of Leuven, Leuven, Belgium). HCT116 cells were cultured in Mccoy’s 5A medium (Thermo Fisher Scientific). HCC827, H1299, SGC7901, and AsPC-1 cells were maintained in RPMI 1640 medium (Corning, Corning, NY, USA). Other cell lines were cultured in DMEM (Corning). All culture media were supplemented with 10% heat-inactivated fetal bovine serum (Thermo Fisher Scientific) and 1% penicillin/streptomycin (Millipore).

### Transient transfection and drug treatment

Cells were transiently transfected with X-Treme Gene HP DNA transfection reagent (Roche) according to manufacturer instructions. For treatments with inhibitors, cells were seeded onto 24-well plates and incubated overnight with indicated compounds before analysis.

### Lentivirus production and cell infection

HEK293T cells were seeded onto 10-cm dish and cotransfected with lentivirus transfer vectors (pCDH-CMV vector carrying AXL, AXL-ICD, AXL-K567R, Flag-AXL) and pPACK Packaging Plasmid Mix (Systems Biosciences) according to manufacturer instructions. Culture medium was concentrated with PEG-*it* precipitation solution (Systems Biosciences). Pseudoviral particles were suspended in prechilled PBS, portioned into aliquots, and stored at −80°C. For infection of HEK293T cells, MEFs, and HCC827 cells, concentrated virus, together with polybrene (8 μg/ml; Sigma-Aldrich), was used. After 48 h transduction, clones that stably overexpressed AXL were screened with 2.5 μg/ml puromycin for 1 wk. Protein expression levels were evaluated by Western blot.

### Small interfering RNA

Panc-28 and HEK293T cells were incubated for 24 h with serum-free Opti-Mem (Thermo Fisher Scientific), 1.5 μl Lipofectamine 3000 (Thermo Fisher Scientific), and 20 pmol small interfering RNA (siRNA) (Genepharma, Shanghai, China) that targeted ADAM10 [siADAM10#1: 5′–AGACAUUAUGAAGGAUUAUTT–3′ and siADAM10#2: 5′–GAC AUUUCAACCUACGAAU–3′ (25, 26)], TACE [TACE#1: 5′–CCAGGGAGGGAAA UAUGUCAUGUAU–3′ and TACE#2: 5′–GAGGAAAGGAAAGCCCUGUACAGUA–3′ (27)], [β-secretase (BACE)#1: 5′–GCUUUGUGGAGAUGGUGGA–3′ and BACE#2: 5′–UGGACUGCAAGGAGUACAA–3′ (28)], or negative control (random sequence, 5′-UUCUCCGAACGUGUCACGU–3′). Cells were then further cultured in normal conditions as previously described. After 48 h post-transfection with siRNA, C-terminal Flag-tagged AXL was transiently transfected into HEK293T cells for an additional 24 h. All cells were then treated with 10 μM DAPT or DMSO for 16 h before mRNA or protein analysis at 72 h post-transfection with siRNA. Knockdown efficiency was confirmed by quantitative PCR and/or Western blot. For mRNA analysis, first-strand cDNA was transcribed from 1 μg total RNA by using ReverTra Ace kit (Toyobo) with Oligo(dT)_20_ primers according to manufacturer instructions, then quantified by real-time PCR with Hieff quantitative PCR SYBR Green Master Mix (Yeasen, Shanghai, China) and StepOnePlus Real-time PCR System (Applied Biosystems, Carlsbad, CA, USA). Quantification of gene expression was calculated by normalization to *GAPDH* using the 2^− ΔΔ^*^Ct^* method (29). Primers for specific targets are as follows: ADAM10 (forward: 5′–CAGACTTCTCCGGAATCCGTAA–3′; reverse: 5′–TGGGAAACGGAAAGGATTTG–3′), TACE (forward: 5′–ACCACCTGAAGAGCTTGTTCATC–3′; reverse: 5′–TTCCCCTCTGTGTA–3′), BACE1 (forward: 5′–ACCAACCTTCGTTTGCCCAA–3′; reverse: 5′–TCTCCTAGCCAGAAACC ATCAG–3′), and GAPDH (forward: 5′–GAAGGTGAAGGTCGGAGTC–3′; reverse: 5′–GAAGATGGTGATGGGATTT–3′).

### NF-κB luciferase reporter assay

A consensus 5× κB response element (5′–GGGAATTTCCGGGGACTTTCCGGGAATTTCCGGGGACTTTCCGGGAATTTCC–3′) was cloned into pGL3 Basic vector (Promega, Madison, WI, USA) with *Kpn*I and *Bgl* II using primers 79–80 (NF-κB-Luc; Table 1 and Supplemental Table 1). Then, NF-κB firefly luciferase reporter was cotransfected into HEK293T cells in the presence or absence of AXL-ICD for 24 h. pGMLR-TK *Renilla* luciferase reporter (Genomeditech, Shanghai, China) was also cotransfected into the indicated cell as an internal control. After 48 h post-transfection, cells were lysed and luciferase activity was sequentially measured according to the dual luciferase activity assay kit (Promega).

### Subcellular fractionation

Cells were grown in a 10-cm dish until 90% confluency, washed twice with cold PBS, scraped and transferred to 1.5-ml Eppendorf tubes, and centrifuged at 500 *g* for 5 min. Cells were then fractionated into nuclear and cytoplasmic fractions according to manufacturer instructions with the NE-PER Nuclear and Cytoplasmic Extraction Kit (Thermo Fisher Scientific).

### Immunoprecipitation

Immunoprecipitation of sAXL was carried out by using anti-DYKDDDDK affinity gel (Biotool). Culture medium from cells that overexpressed N- and C-terminal Flag-AXL was collected and incubated overnight at 4°C with anti-DYKDDDDK affinity gel. Beads were then washed with Tris-buffered saline and bound proteins were eluted with 2× sample buffer at 95°C for 5 min and further analyzed by Western blot.

### Detection of phosphorylated AXL

HEK293T cells were transiently transfected with AXL-FL-WT and AXL-FL-K567R mutant before incubation with DAPT for an additional 16 h. Cells were then lysed with immunoprecipitation buffer [25 mM Tris, 150 mM NaCl, 1 mM EDTA, 1% NP-40, 5% glycerol, 1 mM Na_3_VO_4_, 1 mM PMSF, 1 mM NaF, and 1× protease inhibitor cocktail (Roche); pH 7.4]. Whole-cell lysate was quantified by using a BCA assay kit (Thermo Fisher Scientific). Approximately 500 μg proteins was incubated with AXL (C-20) Ab, which was cross-linked with the protein G-agarose (Roche) by disuccinimidyl suberate to avoid the interference of the heavy chain of the Ab with the detection of AXL-ICD in the immunoprecipitated samples (Pierce Cross-link IP Kit; 26147). Eluted AXL-CTF and AXL-FL were resolved by SDS-PAGE on a 10% Tris-glycine gel and the membrane was blotted with anti-pY (4G10, 1:1000; Millipore) or anti-Flag Ab (Abmart).

### Protein extraction, Western blotting, and quantification

Cells were first lysed in HEPES buffer (pH 7.0) that contained 1% NP-40 or Glo Lysis Buffer (Promega) with protease inhibitor cocktail (Roche). Next, ∼20–60 μg of total proteins (as determined by BCA assay kit) was resolved by PAGE on a 10% Tris-glycine SDS-gel, transferred onto a 0.2-μm PVDF membrane (Bio-Rad, Hercules, CA, USA), and probed with Abs as indicated in the figures.

### Localization study

For the AXL-ICD localization study, HEK293T cells that were grown on coverslips were transiently transfected with AXL-EGFP mutants. After 24 h, cells were fixed with 4% paraformaldehyde, nuclei were stained using 6 μg/ml DAPI, and images were captured using a Nikon-A1 confocal imaging system with a ×63 oil-immersion objective (Nikon, Tokyo, Japan). Nuclear localization of AXL-ICD was estimated by measuring the percentage of cells with a nuclear GFP signal *vs*. all GFP^+^ cells.

### Immunofluorescence

HCC827 cells that stably expressed empty vector, AXL-FL, and AXL-ICD were grown on coverslips, fixed with 4% paraformaldehyde in PBS, permeabilized in 0.02% Triton X-100, and blocked with 1% bovine serum albumin for 1 h at room temperature before overnight incubation at 4°C with Flag Ab. Cells were then incubated with Alexa Fluor 546 anti-mouse secondary Ab (Thermo Fisher Scientific) and stained with DAPI. Images were collected according to the previously described procedure for localization study.

### Purification of γ-secretase, AXL-CTF-Flag, and C100-Flag

γ-Secretase was purified from S-20 cells as previously described (30). Recombinant substrates AXL-CTF (*i.e.*, AXL_(428-527)_-Flag and AXL_(445-527)_-Flag, as well as human APP-C100-Flag) were overexpressed in *Escherichia* *coli* and purified according to a previously described procedure (31).

### *In vitro* purified γ-secretase activity assay

*In vitro* γ-secretase activity assays were performed with purified γ-secretase as previously described (32).

### Cell growth and cell viability assay

To assay erlotinib resistance, 3000 cells/well were seeded onto 96-well plates. After 24 h, medium was replaced with fresh growth medium (100 μl/well) that contained the indicated concentrations of erlotinib or DMSO in sextuples, and cultures were incubated for 72 h. Cell viability was assessed by using the CellTiter96 Aqueous One Solution Cell Proliferation Assay (Promega). Each experiment was repeated twice.

### Statistical analysis

Densities of bands detected by Western blot were quantified with the ImageJ software (National Institutes of Health, Bethesda, MD, USA) and expressed as a percentage of control. Statistical analysis was performed on raw data for each group by Student’s *t* test or ANOVA. A value of *P* < 0.05 was considered statistically significant.

## RESULTS

### AXL is processed by γ-secretase in several cancer cell lines

**Figure 1.**
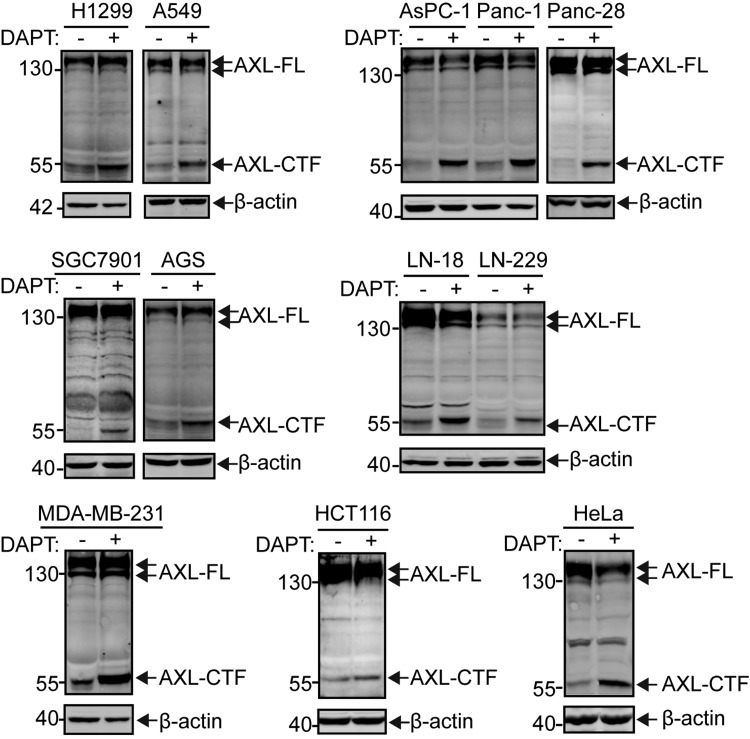
γ-Secretase–dependent processing of AXL in 7 types of cancer cell lines. Pharmacological inhibition of AXL proteolysis in cancer cell lines. DAPT, a well-known inhibitor of γ-secretase, was used at 10 μM to treat in 24-well plates NSCLC cells (H1299 and A549), pancreatic cancer cells (AsPC-1, Panc-1, and Panc-28), gastric cancer cells (SGC7901 and AGS), glioma cells (LN-18 and LN-229), breast cancer cells (MDA-MB-231), colon cancer cells (HCT116), or cervical carcinoma cells (HeLa). After 24-h treatment, cells were collected and lysed in 1% NP-40–HEPES buffer, and equal amounts of protein (normalized by BCA) were loaded onto a 10% Tris-glycine SDS gel and blotted for AXL-FL and AXL-CTF by using a C-terminal Ab (C-20; Santa Cruz Biotechnology). Levels of β-actin were used as an equal loading control. All blots are representative results of at least 2 independent experiments.

To investigate whether AXL can be cleaved by γ-secretase, we first treated various types of cancer cell lines with DAPT, a known inhibitor of γ-secretase (33). In the presence of 10 μM DAPT, a concentration at which the activity of γ-secretase is blocked, we observed a specific accumulation of a ∼55-kDa AXL-CTF in all types of cancer cells tested (**Fig. 1**)—that is, NSCLC cells (H1299 and A549), pancreatic cancer cells (AsPC-1, Panc-1, and Panc-28), glioma cells (LN-18 and LN-229), gastric cancer cells (SGC7901 and AGS), breast cancer cells (MDA-MB-231), colon cancer cells (HCT116), and human cervical carcinoma cells (HeLa). The 55-kDa AXL-CTF fragment, as detected by C-terminal AXL Ab (C-20), corresponds to the size of the AXL cleavage product generated by α-secretase (22). AXL-CTF is a direct substrate for γ-secretase and, consequently, accumulates upon treatment with DAPT. The cleavage event of AXL in these cancer cells seems to be frequent, as reflected by the substantial amount of AXL-CTF generated from AXL-FL in DAPT-treated samples (Fig. 1). Taken together, these data suggest that the proteolytic cleavage of AXL by γ-secretase is widespread across a variety of cancer cells that exhibit high AXL expression levels.

### AXL is sequentially cleaved by α-secretase and presenilin/γ-secretase

**Figure 2.**
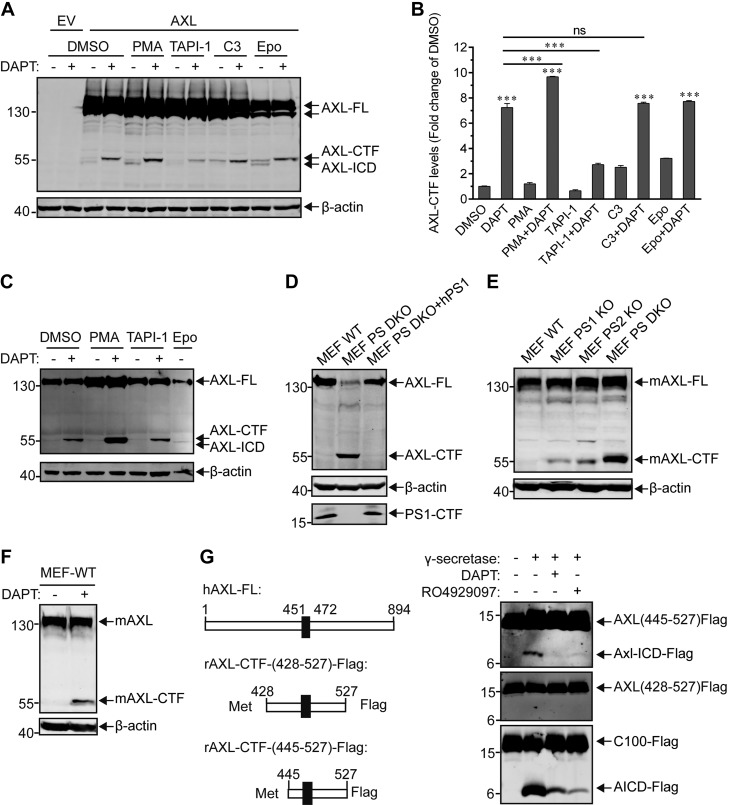
Pharmacological and genetic tools uncover the sequential cleavage of AXL mediated by α- and γ-secretases. *A*, *C*) HEK293T cells either transiently (*A*) or stably (*C*) expressing AXL-FL were treated overnight with DMSO (final concentration, 0.3% w/v), 100 ng/ml PMA, 20 μM α-secretase inhibitor TAPI-1, 5 μM BACE1 inhibitor C3, or 200 nM Epo in the presence or absence of 10 μM DAPT. Cells were then lysed, and whole-cell extracts were separated by PAGE on a 10% Tris-glycine SDS gel for detection of AXL-FL and AXL-CTF with an anti-Flag Ab (Abmart). β-Actin was used as an equal loading control. *B*) Densitometric estimation of the relative levels of AXL-CTF in panel *A*. AXL-CTF levels were quantified by using ImageJ software and expressed as fold-change of DMSO controls. Data are expressed as means ± sem of triplicate measurements; the statistical analyses between the treated and DMSO groups, or between other lined groups are shown with asterisks (*n* = 3). ***P < 0.001, 1-way ANOVA with Bonferroni's multiple comparisons test. *D*) Genetic ablation of γ-secretase activity in PS-DKO MEFs prevented cleavage of AXL. Cell lysates of WT MEFs, PS-DKO MEFs, or PS-DKO MEFs expressing WT hPS1 (PS DKO + hPS1), all of which stably express hAXL-Flag, were probed for hAXL-FL or hAXL-CTF by using an anti-Flag Ab for PS1-CTF (ab76083; Abcam) or β-actin. *E*) Endogenous AXL cleavage was disrupted in MEFs in PS1-KO, PS2-KO, or PS-DKO cells. *F*) Endogenous mouse AXL cleavage was blocked by the γ-secretase inhibitor DAPT (10 μM). Whole-cell extracts were probed with anti-AXL (C-20) antibody (*E*, *F*). Levels of β-actin were used as loading controls. *G*) Recombinant AXL-CTF is directly cleaved in a cell-free activity assay using purified γ-secretase (32). Purified recombinant substrates APP-C100-Flag, AXL-CTF_(428-527)_-Flag, or AXL-CTF_(445-527)_-Flag were incubated with purified γ-secretase at 37°C for 4 h in the presence or absence of DAPT (10 μM) or RO4929097 (100 nM). Enzymatic reactions were stopped by adding 0.5% SDS, and the resulting cleavage products were separated on a 16% Tricine-SDS gel and detected with an anti-Flag Ab (right panel). Schematic diagrams of recombinant AXLs (left). The black box (residues from 451 to 472) represents the transmembrane domain of hAXL. All blots are representative results of at least 2 independent experiments. EV, empty vector; ns, no significance.

Next, we employed pharmacological and genetic tools to further investigate the biochemical cleavage mechanism for AXL. First, HEK293T cells were transiently transfected with an expression plasmid that harbored a Flag-tagged human AXL (hAXL). Cells were then treated with either PMA, a known activator of α-secretase, or various α- or β-secretase inhibitors in the presence or absence of γ-secretase inhibitor DAPT (**Fig. 2*A*, *B***). When compared with DMSO treatments, PMA increased the generation of AXL-CTF, as demonstrated by the more intense AXL-CTF band detected after DAPT treatment (Fig. 2*A*, *B*). A similar observation was made in HEK293T cells that stably expressed hAXL (Fig. 2*C*). AXL-CTF was also found to decrease upon treatment with TAPI-1 (Fig. 2*A*–*C*), an α-secretase inhibitor. In contrast, no significant reduction of AXL-CTF was observed upon treatment with C3, a β-secretase inhibitor, compared with DMSO (Fig. 2*A*, *B*). These data strongly indicate that AXL is processed by α-secretase, but not β-secretase, before γ-secretase cleavage. Moreover, an additional protein migrating just below AXL-CTF was observed in the presence of the proteasome inhibitor Epo (Fig. 2*A*). This protein disappeared after treatment with DAPT, which indicated that it corresponds to γ-secretase–dependent AXL-ICD (Fig. 2*A*).

Second, we used PS-DKO cells to further investigate AXL as a candidate substrate of γ-secretase (34, 35). When WT and PS-DKO MEFs were transduced with lentiviruses that carried C-terminal Flag-tagged AXL, a clear accumulation of hAXL-CTF appeared in PS-DKO MEFs (Fig. 2*D*), which indicated that PS1, PS2, or both are indeed required to process AXL. Furthermore, loss of AXL-CTF cleavage was rescued by transfecting PS-DKO MEFs with human PS1 (Fig. 2*D*). We also found that levels of endogenous AXL-CTFs were elevated in PS1-KO, PS2-KO, and PS-DKO MEFs compared with WT MEFs, which indicated that mouse AXL can be cleaved by either PS1 or PS2 (Fig. 2*E*). Further supporting the latter, the proteolytic cleavage of mouse AXL can be prevented by DAPT (Fig. 2*F*).

Finally, to confirm that AXL-CTF can be directly cleaved by γ-secretase, we generated recombinant AXL-CTFs of different lengths, namely AXL_(428-527)_ and AXL_(445-527)_, that contain the entire AXL transmembrane domain (TMD) and parts of the cytosolic and extracellular domains (Fig. 2*G*, left), and tested them in an *in vitro* activity assay performed with purified γ-secretase. Results demonstrated that AXL_(445-527)_ could be directly cleaved by γ-secretase, leading to the formation of an ∼8-kDa AXL-ICD product (Fig. 2*G*, upper right). Furthermore, cleavage was totally blocked by DAPT or RO4929097, 2 known γ-secretase inhibitors that substantially reduced cleavage of recombinant APP-CTF, another γ-secretase substrate (APP-C100; Fig. 2*G*, bottom right). In contrast, AXL_(428-527)_, the longer form of recombinant AXL-CTF, could not be processed by γ-secretase (Fig. 2*G*, middle right). These results indicated that γ-secretase had a higher affinity for AXL-CTF with a short ectodomain, suggesting that the α-secretase cleavage site may be located near the AXL residue Ala445.

### Regulated intramembrane proteolysis of endogenous AXL occurs in various types of cancer cells

**Figure 3.**
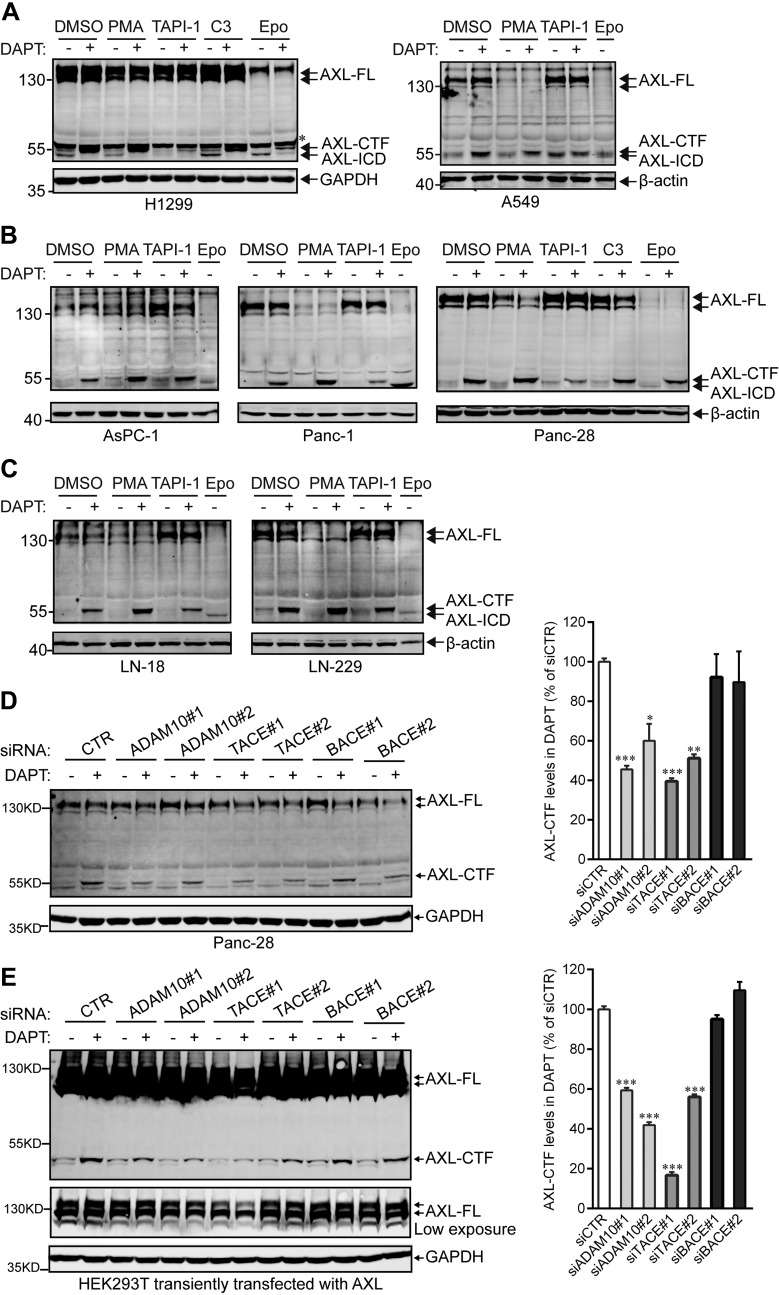
Endogenous AXL in cancer cells is sequentially processed by α- and γ-secretases. *A*–*C*) NSCLC cell lines (H1299 and A549; *A*), pancreatic cancer cell lines (AsPC-1, Panc-1, and Panc-28; *B*), and glioma cell lines (LN-18 and LN-229; *C*) were treated overnight with DMSO, 100 ng/ml PMA, 20 μM TAPI-1, 5 μM C3, or 200 nM Epo in the presence or absence of 10 μM DAPT. Cell lysates were collected for detection of AXL-FL, -CTF, or -ICD using the C-20 Ab targeting the C terminus of AXL. *D*, *E*) α-Secretases ADAM10 and TACE are in charge of the production of AXL-CTF in Panc-28 (*D*) and HEK293T (*E*) cells. Panc-28 and HEK293T cells were transfected with the indicated siRNA in the absence and presence of Flag-tagged AXL-FL, respectively, before the 16-h incubation with DAPT (for details, see Materials and Methods). Cells were then lysed to analyze AXL-FL and AXL-CTF by Western blotting using an anti-AXL Ab (B-2) and an anti-Flag Ab for Panc-28 and HEK293T cells, respectively. AXL-CTF levels in DAPT-treated groups were quantified and expressed as the percentage of control siRNA (siCTR). Data are presented as means ± sem (*n* = 3). Knockdown efficiencies of siRNAs for ADAM10, TACE, and BACE were validated by quantitative RT-PCR and/or Western blots in these samples, and data are shown in Supplemental Fig. 1. GAPDH and β-actin were used as equal loading controls. Asterisk indicates nonspecific band. All blots were performed at least twice, and a representative experiment is shown. *P < 0.05, ***P < 0.01, ***P < 0.001, 1-way ANOVA with Bonferroni's multiple comparisons test.

To determine whether endogenous AXL in cancer cells shares a common cleavage mechanism with exogenously expressed AXL, we treated 3 types of cancer cell lines—lung cancer cells (H1299 and A549; **Fig. 3*A***), pancreatic cancer cells (AsPC-1, Panc-1, and Panc-28; Fig. 3*B*), and glioma cells (LN-18 and LN-229; Fig. 3*C*)—with PMA, as well as with various secretase/protease inhibitors. Similar to the findings depicted in Fig. 2*A–C*, endogenous AXL also underwent sequential cleavage by α- and γ-secretases (Fig. 3). In addition, siRNA genetic knockdown of α- or β-secretase (Fig. 3*D*, *E*, and Supplemental Fig. 1), as well as ablation of PS1 or PS2 (Fig. 2*E*), the catalytic subunit of γ-secretase, confirmed that α-secretase (rather than β-secretase) and γ-secretase are in charge of the cleavage of AXL (Fig. 3*D*, *E*, and Supplemental Fig. 1). Of interest, Epo treatments caused decreased levels of AXL-FL associated with increased levels of AXL-ICD, the end product of RIP (Fig. 3*A*–*C*). As expected, Epo-dependent AXL-ICD production was prevented with DAPT (Fig. 3*A*, H1299; Fig. 3*B*, Panc-28). Altogether, these data may suggest that cellular cleavage of endogenous AXL could be stimulated by preventing the proteasomal degradation of AXL-FL.

### Determination of AXL α- and γ-secretase cleavage sites

**Figure 4.**
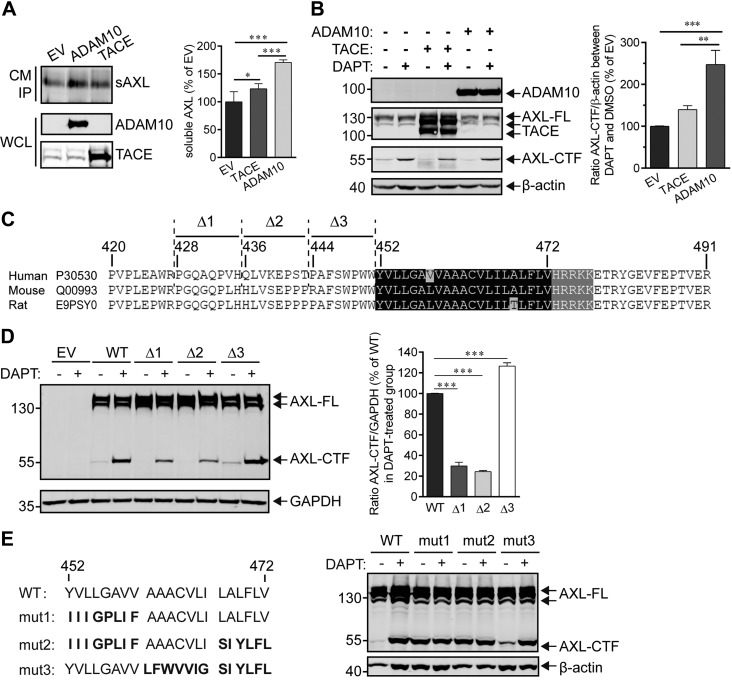
Determination of α- and γ-secretases cleavage sites in AXL. *A*) ADAM10 is the major α-secretase responsible for cleavage of the AXL ectodomain. HEK293T cells that stably expressed N- and C-terminal Flag-tagged AXL-FL were transiently transfected with ADAM10 or TACE for 48 h, and the corresponding culture medium (CM) was then collected for immunoprecipitation by using an anti-Flag affinity resin. Proteins bound to the resin were eluted in 2× sample buffer and subjected to Western blot analysis for detection of sAXL (anti-Flag Ab), ADAM10 (anti-Myc Ab), or TACE (anti-Flag Ab). The densitometric estimation of sAXL was performed by using ImageJ software, and levels of sAXL are expressed as a percentage of the empty vector (EV) control group. Data are presented as means ± sem (*n* = 5). Significance value was estimated using 1-way ANOVA with Bonferroni's multiple comparisons test. *P < 0.05, ***P < 0.001. *B*) HEK293T cells expressing the Flag-tagged AXL-FL were transiently transfected with ADAM10, TACE, or EV for 24 h. Cells were then incubated for 24 h with DMSO (control) or DAPT to induce AXL-CTF accumulation, which reflects the shedding activity of α-secretase on AXL, and cell lysates were collected and subjected to Western blot to analyze AXL-CTF (anti-Flag Ab), mTACE-Flag (anti-Flag Ab), or ADAM10 (Myc Ab) levels. The relative levels of AXL-CTF were quantified by ImageJ software and the ratios between the DAPT- and DMSO-treated groups were expressed as the percentage of the EV group. Data are presented as means ± sem (*n* = 4). **P < 0.01, ***P < 0.001, 1-way ANOVA with Bonferroni's multiple comparisons test. *C*) Amino acid sequence alignment of the juxtamembrane domains of human, mouse, and rat AXL. Δ1, Δ1, or Δ3 refers to the deleted regions in hAXL mutants. The black shaded region is the TMD, and the gray area is the conserved basic amino acid domain in the cytoplasmic region. *D*) Putative AXL cleavage sites in α-secretase. HEK293T cells transiently expressing WT or mutant AXL [*i.e.*, Δ1 (^428^PGQAQPVH^435^-deletion), Δ2 (^436^QLVKEPST^443^-deletion), or Δ3 (^444^PAFSWPWW^451^-deletion)] were treated with DAPT at 10 μM, and cell lysates were analyzed for AXL-FL and AXL-CTF (anti-Flag). AXL-CTF or GAPDH bands in the blot (left) were quantified by using ImageJ software. The ratio between AXL-CTF and GAPDH was calculated and normalized to the WT group (right). Results are expressed as means ± sem (*n* = 4). ***P < 0.001, 1-way ANOVA with Bonferroni's multiple comparisons test. *E*) Putative γ-secretase cleavage sites in AXL. HEK293T cells were transiently transfected with WT or mutants AXL (mut1-3; left), in which the TMD was substituted with sequences of the InsR (bold fonts; left), which has previously been used to identify the cleavage sites for γ-secretase (37). Cells were then treated overnight with 10 μM DAPT before analysis of AXL-FL and -CTF (anti-Flag; right). The immunoblot shows representative results of at least 2 independent experiments. β-Actin or GAPDH serve as the loading controls.

Because AXL could be sequentially cleaved by α- and γ-secretases in cells and in cell-free γ-secretase activity assays performed with purified enzyme, we next investigated the type of α-secretase that participates in this process and identified the α- and γ-secretase cleavage sites in AXL. To determine whether ADAM10 or TACE was involved in α cleavage, we transfected HEK293T cells that stably expressed hAXL with plasmids that harbored ADAM10 or TACE genes (**Fig. 4*A***), and examined the production of extracellular sAXL and intracellular AXL-CTF, the two α-secretase cleavage products. Results showed an increase in sAXL and AXL-CTF in both ADAM10- and TACE-transfected groups (Fig. 4*A*, *B*); however, whereas transfection with ADAM10 caused ∼170 and ∼230% increases in sAXL and AXL-CTF, respectively, transfection with TACE only increased shedding by ∼20% compared with empty vector (Fig. 4*A*, *B*). Together, these data show that the main sheddase activity for AXL can be attributed to ADAM10 (19, 23). Next, we aimed at determining the AXL α-secretase cleavage sites by using a site-directed mutagenesis approach. Shedding was decreased by ∼60% in both AXL mutants Δ1 (^428^PGQAQPVH^435^-deletion) and Δ2 (^436^QLVKEPST^443^-deletion) compared with AXL WT (Fig. 4*C*, *D*). In contrast, substrate shedding was maintained in the mutant Δ3 (^444^PAFSWPWW^451^-deletion; Fig. 4*C*, *D*). These data suggest that the region between aa 428 and 444 may contain the cleavage site for α-secretase. Several point mutations (L437, V438 alone or in combination) had no effect on AXL shedding (Supplemental Fig. 2), which reflected a previously reported loose sequence specificity for α-secretase cleavage (36).

To characterize the γ-secretase cleavage site, we substituted parts of the TMD of AXL with the corresponding TMD sequence of the insulin receptor (InsR), whose sequence has been previously used to identify the cleavage site of a γ-secretase substrate (37). Mutants 1 and 2 (mut1 and mut2; Fig. 4*E*) were not processed by γ-secretase as AXL-CTF did not accumulate upon treatment with DAPT (Fig. 4*E*). In contrast, mut3 and mut4 were still cleaved by γ-secretase (Fig. 4*E* and Supplemental Fig. 3*A*), whereas partial mutations of the mut1 region (the first 8 aa of the N terminus in TMD) or V472 mutant also allowed substrate processing by γ-secretase (Supplemental Fig. 3*B*, C). Collectively, these results suggested that the common substituted domain in mut1 and mut2 [that is, the YVLLGAVV (from 452 to 459)] is the essential cleavage region for γ-secretase, whereas minor modifications of the TMD do not affect γ cleavage. In addition, mouse and rat AXL are highly but imperfectly conserved in the TMD relative to hAXL (Fig. 4*C*) and are still sensitive to DAPT treatment (Supplemental Fig. 3*D*), which implies that the sequence specificity for γ-secretase is loose (38). In sharp contrast, MERTK and TYRO3, the other members of the TAM family whose TMDs are largely different from AXL, are very poorly or not at all processed by γ-secretase (Supplemental Fig. 3*E*).

### Abolishment of the kinase activity of AXL accelerates its proteolysis by α-secretase

**Figure 5.**
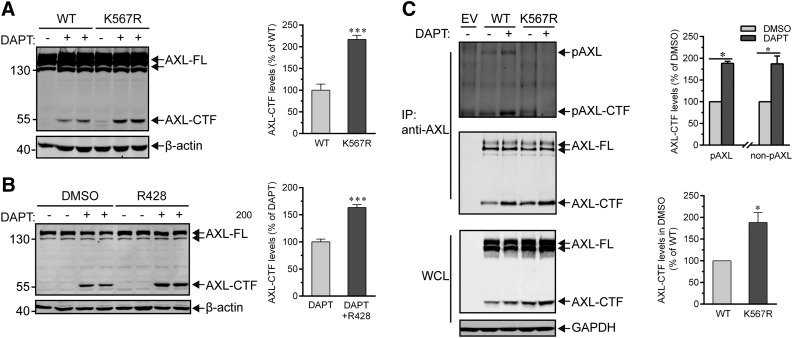
Abolishment of the kinase activity of AXL accelerates its proteolysis by α-secretase. *A*) AXL kinase activity is not required for the regulated proteolysis of AXL. HEK293T cells were transiently transfected with AXL-FL WT or K567R mutant for 24 h and further incubated with 10 μM DAPT before analysis by Western blot of AXL-FL and -CTF protein levels (anti-Flag; left). AXL-CTF levels of DAPT-treated cells transfected with AXL-FL WT or K567R were quantified and expressed as a percentage of WT values (right). Data are shown as means ± sem (*n* = 4). ***P < 0.001, unpaired Student’s *t* test. *B*) The processing of AXL is increased by an AXL kinase inhibitor. Panc-28 cells were incubated overnight with DMSO or 150 nM R428, a selective AXL kinase inhibitor, before examining by Western blot the levels of endogenous AXL-FL and AXL-CTF (C-20; left). Levels of AXL-CTF in DAPT-treated cells were quantified as described above and unpaired Student’s *t* test was employed for the analysis (right). Data are shown as means ± sem (*n* = 4). ***P < 0.001. *C*) Phosphorylated or nonphosphorylated AXL-CTF is processed with the same potency by γ-secretase. HEK293T cells expressing WT AXL-FL or the mutant AXL-FL K567R mutant were lysed and equal amounts of whole-cell lysates (WCL) were precipitated with the disuccinimidyl suberate–cross-linked AXL (C-20) Ab. Eluted proteins were then resolved by PAGE on a 10% Tris-glycine SDS gel and blotted with anti-pY (4G10) and anti-Flag Ab for phosphorylated (top) and total (middle) AXL-FL or AXL-CTF, respectively. Relative levels of phosphorylated AXL-CTF [the anti-pY blot for AXL-wild-type (WT AXL-FL); top] and nonphosphorylated AXL-CTF (the anti-Flag blot for AXL-K567R mutant; middle) in the immunoprecipitated (IP) samples were quantified by densitometry and expressed as percentage of DMSO controls and shown as means ± sem (*n* = 3). Relative levels of AXL-CTFs in the DMSO groups from the anti-Flag blot (middle) were additionally displayed (AXL-WT, 100%; means ± sem; *n* = 3). *P < 0.05, paired Student’s *t* test. Immunoblots show representative results of at least 3 independent experiments. β-Actin or GADPH serves as the loading control.

To explore whether the kinase activity of AXL influences its cleavage process mediated by α- or γ- secretases, we mutated the AXL ATP-binding site K567 to Arg (K567R, kinase-dead AXL) (39) and transiently transfected HEK293T cells with this mutant to examine its ability to be processed. Results showed that overall AXL-CTF production from mutant K567R was increased by ∼200% compared with WT substrate (**Fig. 5*A***). This indicates that the kinase activity of AXL is not necessary to its proteolysis. Consistent with this observation, we found that R428, a selective inhibitor of AXL kinase (40), could facilitate α cleavage and result in a ∼150% increase of AXL-CTF in Panc-28 cells (Fig. 5*B*). Increased cleavage of nonphosphorylated AXL-FL (K567R mutant) is likely not caused by intramembrane proteolysis mediated by γ-secretase, as phosphorylated AXL-CTF (pAXL-CTF) and non–pAXL-CTF displayed a similar capacity to be processed by γ-secretase (Fig. 5*C*). However, the levels of non–pAXL-CTF (K567R) in DMSO-treated samples were found to be significantly higher than the AXL-CTF generated from WT AXL-FL (Fig. 5*C*, bottom and right), which suggests that α-secretase activity was enhanced by the ablation of the activity of AXL kinase (Fig. 5*C*). Supporting this observation, inhibition of AXL kinase activity by R428 has been successfully used to overcome cancer cell resistance induced by TKIs, an acquired chemoresistance that is attributed to a reduced proteolytic shedding by α-secretase (23). Taken together, these data demonstrated that AXL kinase activity is not required for cleavages mediated by α- or γ-secretase and that inhibition of the activity promotes overall cleavage efficiency for AXL, a phenomenon that is likely a result of the elevated activity of α-secretase.

### AXL-ICD localizes to the nucleus *via* a NLS motif

**Figure 6.**
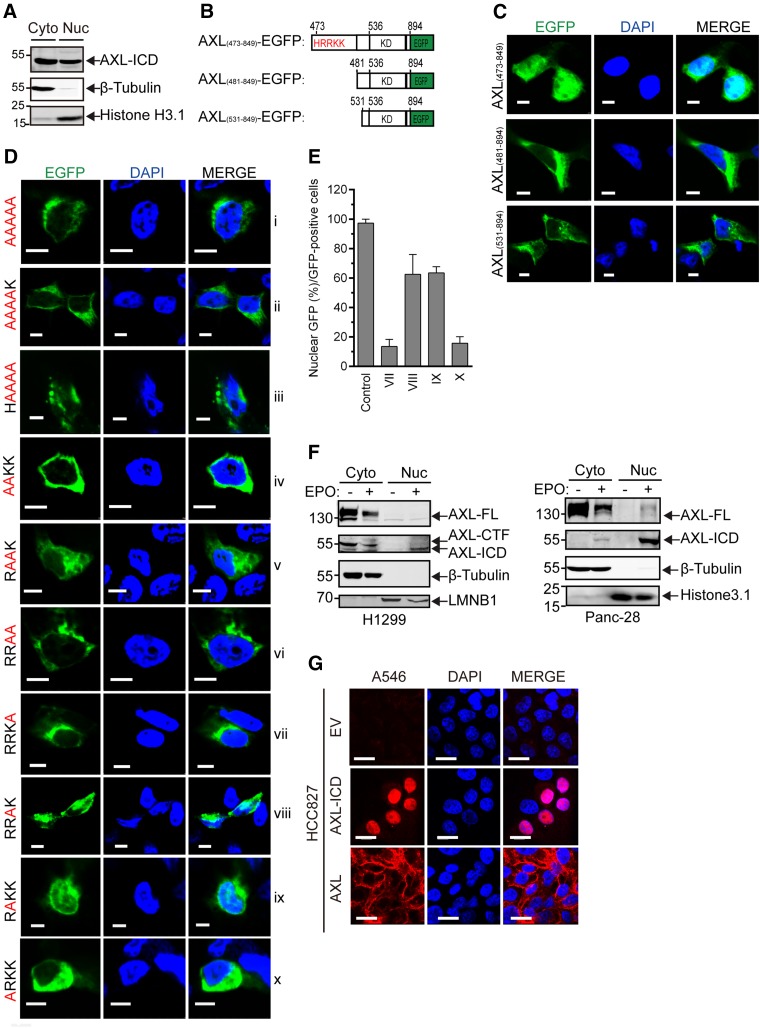
Nuclear localization of AXL-ICD. *A*) Cellular fractionation of cytoplasmic (Cyto) and nuclear (Nuc) fractions shows the translocation into the nucleus of AXL-ICD. HEK293T cells stably expressing AXL-ICD were collected from a 10-cm dish, and the cytosolic and nuclear fractions were obtained by using the NE-PER Nuclear and Cytoplasmic Extraction Kit. Fractions were subjected to analysis of the AXL-ICD levels by immunoblot with the anti-Flag Ab. β-Tubulin and Histone H3.1 were used as internal controls for cytoplasmic and nuclear fractions, respectively. *B*) Schematic representation of the different AXL-ICD-EGFP fusion proteins used in this study. *C*) AXL-ICD-EGFP translocates into the nucleus. HEK293T cells were transiently transfected for 24 h with AXL_(473-894)_-EGFP (top), AXL_(481-894)_-EGFP (middle), or AXL_(531-894)_-EGFP (bottom), and fluorescent images of EGFP fusion proteins (green) were taken using a Nikon-A1 confocal microscope. The nucleus was counterstained with DAPI (blue). Cyan, colocalization of EGFP fusion proteins (green) and DAPI (blue). Scale bars, 5 μm. *D*) Identification in AXL-ICD of the nuclear localization sequence. HEK293T cells were transiently transfected with different mutants of AXL-ICD_(473-849)_-EGFP [that is, quintuple or quadruple mutants for the ^473^HRRKK^477^ basic motif (*i–iii*), double mutants (*iv–vi*), or single mutants (*vii–x*)]. Corresponding images were obtained as described in panel *C*. Cyan, colocalization of EGFP fusion proteins (green) and DAPI (blue). Scale bars, 5 μm. *E*) Quantification of the percentage of nuclear localization of single mutants of AXL-ICD_(473-849)_-EGFP (*Dvii*–*x*) as well as AXL-ICD_(473-849)_-EGFP (control; *C*). AXL-ICD nuclear localization was evaluated by the percentage of cells with a positive GFP signal in the nucleus among all GFP^+^ cells. Quantification experiments were repeated 3×, and the average percentages are expressed as means ± sem (*n* = 3). *F*) Endogenous AXL-ICD translocates into the nucleus of cancer cells. H1299 (right) or Panc-28 cells (left) in the presence or in the absence of Epo were collected from a 10-cm dish, and cytosolic and nuclear fractions were obtained as previously described (*A*). Levels of AXL-FL, -CTF, or -ICD were analyzed by immunoblot with the C-20 anti-AXL Ab. β-Tubulin was used as an internal control for cytoplasmic fractions, whereas histone H3.1 or LMNB1 were used as controls for nuclear fractions. *G*) Immunofluorescence detection of AXL-ICD in the nucleus. HCC827 cells stably expressing AXL-FL-Flag or AXL-ICD-Flag were sequentially stained with an anti-Flag Ab and Alexa Fluor 546 anti-mouse secondary Ab (red); the nucleus was stained with DAPI (blue). Immunofluorescence images were obtained with a Nikon-A1 confocal microscope. Magenta, colocalization of AXL-ICD (red) and DAPI (blue). Scale bars, 25 μm. EGFP, enhanced-green fluorescent protein; KD, kinase domain.

To determine the fate of AXL-ICD, we asked whether it could translocate into the nucleus, similar to other RTK-ICDs produced by γ-secretase (41). To address this question, we separated the cytosolic and nuclear fractions of HEK293T cells that stably expressed the entire sequence of the AXL ICD (aa residues 473 to 894). As shown in **Fig. 6*A***, a substantial amount of ICD was detected in the nuclear fraction.

To visualize the cellular location of AXL-ICD, we fused its C terminus with EGFP and expressed the fusion protein in HEK293T cells (Fig. 6*B*). A strong nuclear colocalization of AXL-ICD-EGFP with DAPI was found (Fig. 6*C*, top, cyan), which confirmed the translocation of AXL-ICD into the nucleus. To identify a potential NLS in charge of this translocation, we removed the N-terminal sequence of AXL-ICD, which is enriched in His/Lys/Arg basic amino acids (that is, HRRKK) and has the potential to function as a NLS after γ-secretase cleavage (38). Confirming our hypothesis, truncated AXL_(481-894)_ was exclusively localized in the cytoplasm (Fig. 6*C*, middle). Similar observations were made for truncated AXL-ICD with a larger deletion in the N terminus (AXL_(531-894)_; Fig. 6*C*, bottom).

In addition, alanine substitutions of all NLS residues (473-HRRKK-477 into 473-AAAAA-477) completely prevented AXL-ICD from entering the nucleus (Fig. 6*Di*). Similar results were obtained by mutating 4 sequential residues (473-HRRK-476 and 474-RRKK-477; Fig. 6*D**ii*, *iii*). Double mutations of R474/R475 or K476/K477 also completely altered the function of the NLS (Fig. 6*D**iv*, *vi*); however, the double mutation of R475/K476 decreased nuclear localization to a much lesser degree compared with WT control (Fig. 6*D**v*, *C*), which indicated that these 2 residues are likely not crucial for the function of NLS. Indeed, AXL-ICD with a single mutation of R475 or K476 targeted the nucleus more (∼60%; Fig. 6*D**viii*, *ix*, *E*) than did that with single mutations of R474 or K477 (∼20%; Fig. 6, *D**vii,* *x*, *E*). Altogether, our results demonstrate that the HRRKK motif serves as the NLS in charge of the nuclear translocation of AXL-ICD and that residues R474 and K477 play a major role in this function.

Of importance, we have also been able to detect endogenous AXL-ICD in the nuclear fractions of H1299 or Panc-28 cancer cells, whereas AXL-FL was undetected in the nucleus (Fig. 6*F*). In addition, immunofluorescence studies on HCC827 cells that stably expressed AXL-ICD clearly showed that AXL-ICD was enriched in the nucleus (Fig. 6*G*, middle). As expected, AXL-FL was exclusively located at the cell membrane (Fig. 6*G*, bottom), which confirmed that the exposure of the N-terminal NLS sequence released by γ-secretase is indispensable for the nuclear translocation of AXL-ICD.

### Preventing intramembrane cleavage of AXL elevates chemoresistance in NSCLC cells

**Figure 7.**
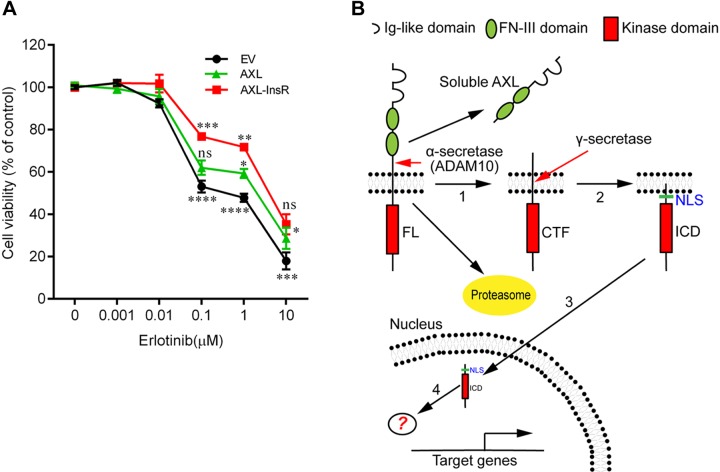
Ablation of intramembrane cleavage of AXL-FL elevates drug resistance in HCC827 cells. *A*) Blockage of AXL cleavage mediated by γ-secretase promotes erlotinib chemoresistance in HCC827 cells. HCC827 cells stably expressing WT AXL-FL (AXL-WT) or the γ-secretase–uncleavable AXL mutant (AXL-InsR), or transfected with the empty vector (EV), were incubated for 72 h with the indicated concentrations of erlotinib, and the cell number was measured by using the CellTiter96 Aqueous One Solution Cell Proliferation Assay. Experiments were independently repeated 2× in sextuples, and 1 representative result is shown. Means at each concentration are given as percentage of the DMSO control. Data are presented as means ± sem (*n* = 6). Statistical analyses were performed by 2-way ANOVA with Bonferroni post-tests. The significance of AXL-InsR compared with AXL-WT is indicated above the red line, whereas the comparison with EV is indicated below the black line, and the significance of AXL-WT and EV is above the green line. *P < 0.05, **P < 0.01, ***P < 0.001, ****P < 0.0001. *B*) Model for the successive proteolytic processing of AXL by α- and γ-secretases. AXL RTK is sequentially cleaved by α-secretase (*1*) and γ-secretase (*2*). Next, AXL-ICD, the cleavage product of the γ-secretase–dependent regulated intramembrane proteolysis, translocates into the nucleus *via* an NLS (3). By interacting with yet unknown intranuclear binding partners (*4*), AXL-ICD potentially modulates the expression of genes (for example, NF-κB–dependent genes) implicated in TAM-dependent signaling pathways. In addition, AXL-FL could be processed *via* the ubiquitin-proteasome proteolytic pathway (51), inhibition of which could accelerate the secretases mediated proteolytic pathway of AXL (*1*, *2*). Ns, no significance.

Overexpression of AXL has been demonstrated to cause drug resistance in lung and breast cancer cells (5, 6, 17). Recently, reduced shedding of AXL has also been implicated in the drug resistance mechanism of TKIs in melanoma (23). As we have demonstrated that intramembrane cleavage of AXL-FL actively occurs in cancer cells (Figs. 1 and 3), we wondered whether the γ-secretase–mediated processing of AXL plays a role in the drug resistance of TKIs. To examine the biological function of the AXL cleavage process, we took advantage of our γ-secretase–uncleavable AXL mutant (Fig. 4*E*, mut1) that we stably expressed in HCC827 cells (HCC827-AXL-InsR, hereafter). Our initial results showed that the IC_50_ of erlotinib in HCC827-AXL-InsR cells (∼6 μM; **Fig. 7*A***) was increased by at least 60-fold over HCC827 cells transduced with empty vector (IC_50_, ∼100 nM; Fig. 7*A*). A similar resistance was observed in A549 and H1299 cells (Supplemental Fig. 4), both of which express high levels of endogenous AXL-FL (Figs. 1 and 3). In addition, it seems that AXL-InsR improves cell viability compared with WT AXL-FL (Fig. 7*A*), which implies that AXL-FL cleavage mediated by γ-secretase may reduce chemoresistance of erlotinib.

## DISCUSSION

The TAM family is the most recently discovered RTK family and plays an essential role in tumor cell survival (5). TAM receptors activate a number of diverse downstream signaling pathways, but the exact molecular mechanisms are not completely understood (42). Canonical RTK signaling is triggered by ligand binding and activation of intracellular kinase cascades *in situ*; however, some RTKs have been demonstrated to be processed by a number of proteases and thus generate intracellular cleavage products (2, 41). In the present study, we systematically characterized the proteolytic process of TAM receptors and found that AXL, but not MERTK or TYRO3, is efficiently and sequentially processed by α- and γ-secretases in 7 types of cancer cells (Figs. 1–4). In addition, we found that AXL-ICD could be transported into the cell nucleus *via* a specific NLS (Fig. 6). Of interest, the γ-secretase–uncleavable AXL-FL mutant caused enhanced chemoresistance compared with WT AXL-FL (Fig. 7*A*), which implied that cleavage driven by γ-secretase may suppress the drug resistance promoted by TKIs. Altogether, our data suggest the existence of a homeostatic balance between the intracellular trafficking and sequential processing of AXL-FL and the cellular localization of cleavage products sAXL/AXL-ICD in cancer cells, a balance that is maintained by α- and γ- secretases and the proteasome (Fig. 7*B*).

AXL-FL is known to be expressed at high levels in several types of cancers, particularly in drug-resistant cancers (5, 6). This is also observed in 7 types of cancer cell lines that were tested in the present study (Fig. 1). In particular, we found that the metabolism of AXL-FL is sensitive to treatment with DAPT in NSCLC cells, which is in agreement with a recent observation that AXL can be degraded in H292 NSCLC cells by presenilin, the catalytic subunit of γ-secretase, as suggested on a single Western blot by the fact that AXL was sensitive to treatment with a γ-secretase inhibitor (43). As we have demonstrated that the abundance of AXL-FL is largely dependent on shedding and intramembrane cleavage by α- or γ- secretases in several types of cancers, it may suggest that reduced proteolytic activities could universally cause the accumulation of AXL-FL in cell membranes. In that regard, proteolytic cleavage of AXL by secretases could potentially play an essentially anti–drug-resistant role in cancer. Supporting the latter hypothesis, a recent study demonstrated in patients with chemoresistant melanoma a reduced proteolytic shedding of RTKs, mainly for AXL but also MET, by α-secretases (23). Consistent with this, we also uncovered that intramembrane cleavage of AXL plays an anti–drug-resistance role in NSCLC cells (Fig. 7*A*). Of interest, γ-secretase inhibitor Semagacestat, a DAPT analog that inhibits the activity of γ-secretase *in vivo*, has been indicated to cause more skin cancers in clinical phase III investigations (44–46). Moreover, cleavage of AXL by RIP seems to be potentiated by the AXL kinase-dead mutant (K567R) or selective AXL inhibitor R428 (Fig. 5*A*, *B*), the latter of which is widely used for reducing chemoresistance caused by overexpression of AXL and is now under clinical phase I and II trial (9). Altogether, these findings support the plausibly defeating role of the AXL shedding and intramembrane cleavage for acquired drug resistance in cancer. Conversely, our results seem to also indicate that AXL is an efficient substrate for α- and γ-secretases. Indeed, a proteomic study had raised the possibility that, other than APP, AXL is an efficient substrate for α-secretase (47).

Of interest, we noticed that intracellular levels of AXL-FL were largely reduced upon treatment with Epo (Figs. 2*A*, *B* and 3). We attributed this reduction to an increased cleavage of AXL-FL by α- and γ-secretases, as the strong reduction of AXL-FL trigged by proteasomal inhibition can be reversed by α-secretase inhibitor TAPI-1 (Supplemental Fig. 5), and a more pronounced AXL-ICD production was observed in Epo-treated cells (Figs. 2*A*, *B* and 3). Taken together, it suggests that proteasome-dependent degradation of AXL-FL is closely coupled with production of ICD (Fig. 7*B*), involving a complex crosstalk between the presenilin- and proteasome-dependent degradation of type I membrane proteins (48). In addition, proteasomal inhibition could be an alternative strategy to reverse the adverse effects of AXL.

Of interest, we also found that AXL-ICD, but not AXL-FL, could translocate into the nucleus *via* a NLS that contained the residue HRRKK (Fig. 6), although considerable amounts of AXL-ICD were retained in the cytoplasm. This trafficking event is dependent on AXL cleavage by γ-secretase, thus exposing the active NLS at the N terminus of the AXL-ICD and inducing its translocation into the nucleus. This is in agreement with the proposal that a potential NLS enriched with Arg/Lys exists in type I membrane proteins, which could be activated by γ-secretase cleavage (38). Elevated levels of AXL-ICD were found in the nucleus after treatment with a proteasome inhibitor (Figs. 2*A*, *B*; 3; and 6*F*). The substantial amount of AXL-ICD present in the nuclei of cancer cells immediately suggests that AXL-ICD could play a role in the nucleus (Fig. 6*F*)—possibly negative regulation of the expression of cancer-related genes. Indeed, we found that AXL-ICD could suppress NF-κB–dependent gene transcription in a luciferase reporter assay (Supplemental Fig. 6), the activation of which is known to be a hallmark for chemoresistance (6, 49). Of interest, Notch-ICD was also reported to directly bind to NF-κB and inhibit its transcriptional activity (50), suggesting that AXL-ICD may function *via* a similar mechanism. However, the precise function of AXL-ICD cannot be defined until the physical binding partner and its downstream regulated genes have been identified (3, 4). Nevertheless, the distribution of AXL across the plasma membrane, cytoplasm, and nucleus suggests that this protein receptor may carry out diverse functions across these different intracellular compartments (Fig. 7*B*).

In summary, we found that AXL can be processed by α- and γ-secretases, thus releasing an AXL-ICD that further translocates into the nucleus. The dynamic processing of AXL-FL and the subcellular localization of AXL cleavage products not only provide new insights into the diverse functions of AXL, but also open potential new strategies for cancer therapy.

## Abbreviations

ADAM: a disintegrin and metalloproteinase
AXL-CTF: AXL C-terminal fragment
AXL-FL: full-length AXL
AXL-ICD: AXL intracellular domain
BACE: β-secretase
DAPT: *N*-[*N*-(3,5-difluorophenacetyl)-L-alanyl]-*S*-phenylglycine *t*-butyl ester
EGFP: enhanced green fluorescent protein
Epo: epoxomicin
GAPDH: glyceraldehyde 3-phosphate dehydrogenase
hAXL: human AXL
ICD: intracellular domain
InsR: insulin receptor
KO: knockout
MEF: mouse embryonic fibroblast
NLS: nuclear localization sequence
NSCLC: non–small-cell lung cancer
PC: phosphatidylcholine
PMA: phorbol 12-myristate 13-acetate
PS1/2: presenilin1/2
PS DKO: presenilin1/2 double-knockout
RIP: regulated intramembrane proteolysis
RTK: receptor tyrosine kinase
sAXL: soluble AXL
TAM: TYRO3, AXL and MERTK
siRNA: small interfering RNA
TACE: tumor necrosis factor α converting enzyme
TAPI-1: TNF-α protease inhibitor 1
TKI: tyrosine kinase inhibitor
TMD: transmembrane domain
WT: wild-type
